# A Personalized Healthcare Monitoring System for Diabetic Patients by Utilizing BLE-Based Sensors and Real-Time Data Processing

**DOI:** 10.3390/s18072183

**Published:** 2018-07-06

**Authors:** Ganjar Alfian, Muhammad Syafrudin, Muhammad Fazal Ijaz, M. Alex Syaekhoni, Norma Latif Fitriyani, Jongtae Rhee

**Affiliations:** 1 U-SCM Research Center, Nano Information Technology Academy, Dongguk University, Seoul 100-715, Korea; 2 Department of Industrial and Systems Engineering, Dongguk University, Seoul 100-715, Korea; udin@dongguk.edu (M.S.); fazal@dongguk.edu (M.F.I.); alexs@dongguk.edu (M.A.S.); norma@dongguk.edu (N.L.F.)

**Keywords:** diabetes, BLE, real-time data processing, classification, forecasting

## Abstract

Current technology provides an efficient way of monitoring the personal health of individuals. Bluetooth Low Energy (BLE)-based sensors can be considered as a solution for monitoring personal vital signs data. In this study, we propose a personalized healthcare monitoring system by utilizing a BLE-based sensor device, real-time data processing, and machine learning-based algorithms to help diabetic patients to better self-manage their chronic condition. BLEs were used to gather users’ vital signs data such as blood pressure, heart rate, weight, and blood glucose (BG) from sensor nodes to smartphones, while real-time data processing was utilized to manage the large amount of continuously generated sensor data. The proposed real-time data processing utilized Apache Kafka as a streaming platform and MongoDB to store the sensor data from the patient. The results show that commercial versions of the BLE-based sensors and the proposed real-time data processing are sufficiently efficient to monitor the vital signs data of diabetic patients. Furthermore, machine learning–based classification methods were tested on a diabetes dataset and showed that a Multilayer Perceptron can provide early prediction of diabetes given the user’s sensor data as input. The results also reveal that Long Short-Term Memory can accurately predict the future BG level based on the current sensor data. In addition, the proposed diabetes classification and BG prediction could be combined with personalized diet and physical activity suggestions in order to improve the health quality of patients and to avoid critical conditions in the future.

## 1. Introduction

Diabetes mellitus, more commonly referred as diabetes, has become a worldwide epidemic. Diabetes is a long-term metabolic disorder in which the blood glucose (BG) level varies and is caused by either insufficient insulin production in the body (Type 1 diabetes, T1D) or by the body’s inability to utilize its produced insulin (Type 2 diabetes, T2D) [1,2,3]. The diagnosis of both T1D and T2D has increased, but the rise has been greater for T2D, which accounts for 90–95% of all cases of diabetes and is a growing epidemic that places a severe burden on healthcare systems, especially in developing countries [4]. Around 4.8 million Koreans (13.7% of the Korean population) aged 30 years or older had diabetes in 2014 [5], while in the US, 30.3 million people of all ages (9.4% of the US population) had diabetes in 2015 [6]. Worldwide, the total number of people with diabetes is projected to rise from 171 million in 2000 to 366 million in 2030 [7]. Ineffectively supervised diabetes prompts serious problems such as cardiovascular diseases, including hypertension and stroke [8]. However, the regular monitoring of the BG level plays a prominent role in reducing and avoiding the complications of diabetes [9,10,11].

Recent advances in information and communication technologies (ICT) along with novel biosensors that can provide real-time monitoring of a patient’s condition offer a new perspective in diabetes management. Diabetic patients can monitor glucose changes with a self-monitoring of blood glucose (SMBG) portable device [12,13,14] as well as continuous glucose monitoring (CGM) sensors [3], and thus they can respond immediately with the appropriate action. The results show that monitoring the glucose levels of patients can provide better control over their condition [14] and improves the performance of diabetes management [15,16]. A glucose monitoring system consisting of sensors, a gateway (smartphone), and a cloud system is the best candidate to improve diabetes management [17]. It collects sensor data from a sensor node attached to the body through a smartphone as a gateway [18]. Communication between the sensor node and the smartphone requires the use of wireless technology as well as low-power operation for the sensor node, and the best candidate for this is Bluetooth Low Energy (BLE) [19,20,21].

Currently, the number of BLE-based wearable sensors, mobile devices (smartphones), and other connected things is increasing significantly. With the shocking amount of sensor data that is produced by a sensor device, this is undeniably the era of big data. Studies regarding the application of big data analytics in healthcare showing positive results have been conducted [22,23,24]. The NoSQL database (DB) is considered as the best solution to handle the massive amounts of sensor data as it provides high performance [25,26,27,28] as well as promise for applications in the healthcare industry [29,30]. Furthermore, due to the increased risk of diabetes, current studies use advanced information technology techniques such as machine-learning algorithms, which can be utilized to predict diabetes based on the current condition of patients and thus can help them discover the risk of diabetes at an early stage [31,32,33,34,35,36]. In addition, machine-learning algorithms could be utilized to predict future BG levels. Several studies have demonstrated BG level prediction, which can help the patient by obtaining a future BG level reading so that preventive alerts can be generated before critical hypo/hyperglycemic events occur [37,38,39,40].

In this paper, we propose a personalized healthcare monitoring system for diabetic patients by utilizing BLE-based sensors and real-time data processing. The latter utilizes Apache Kafka to handle incoming sensor data whereas MongoDB is utilized to store the unstructured sensor data. By utilizing real-time data processing, the vast amount of continuous data (e.g., BG, heart rate, blood pressure, weight, and other personal data) from the BLE-based sensor devices can be handled in real-time. Moreover, the classification algorithm based on a Multilayer Perceptron (MLP) is used to classify the diabetes patient, while Long Short-Term Memory (LSTM) is utilized to predict the BG level.

The remaining organization of this paper is as follows. In Section 2, we present the related works in glucose monitoring, BLE, real-time data processing, and machine learning–based algorithms for monitoring diabetes. Section 3 contains the system design with architecture and its implementation. In Section 4, the results of the study and a discussion are presented. In Section 5, we discuss several limitations and remaining challenges, and finally, concluding remarks are offered.

## 2. Literature Review

### 2.1. Glucose Monitoring and BLE

Diabetes is a chronic disease caused by the absence of insulin secretion (T1D) or defective insulin secretion and action (T2D). For people with diabetes, the BG level exceeds the safe range of 70–180 mg/dL [1,2,3]. Diabetes may be diagnosed based on plasma glucose criteria of either fasting plasma glucose (FPG ≥ 126 mg/dL) or 2-h plasma glucose (2-h PG ≥ 200 mg/dL) levels during a 75-g oral glucose tolerance test (OGTT) or an A1C test (A1C ≥ 6.5%) criteria. Furthermore, in a patient with classic symptoms of hyperglycemia or a hyperglycemic crisis, diabetes may be diagnosed when the random plasma glucose level ≥ 200 mg/dL [9]. Diabetes has been associated with many medical conditions, including hypertension and stroke [8]. Although there is no cure for diabetes, BG monitoring combined with appropriate medication can enhance treatment efficiency, alleviate the symptoms, and diminish complications. The results of studies have shown that the appropriate management of the disease can control and prevent many complications [9,10,11]. A healthy diet, physical exercise, and drug administration are suggested for T2D, while daily insulin administration is required for T1D.

It is important to monitor the glucose levels of patient with diabetes, as it can provide better control over their condition [14]. Glucose monitoring can be utilized to optimize patient treatment strategies such as the effect of medications, exercise, and/or diet [41]. The SMBG device allows the patient to monitor the glucose change as well as to respond immediately with the appropriate action. SMBG utilizes glucose sensors based on electrochemical methods [12] and provides patients with the ability to self-monitor BG levels so as to manage insulin levels. The most widely used SMBG portable device involves sampling blood from a finger via pricking (finger pricking), after which the blood sample is analyzed using in vitro methods such as a test strip and/or a glucose meter [13,14]. A report by the American Diabetes Association (ADA) revealed that the standard therapy for diabetes management consists of three or more SMBG measurements per day, each acquired by a finger prick test [2]. In the majority of cases, SMBG time series are collected by the patient and then analyzed and interpreted by the physician during periodic visits, e.g., every two/four months, after which the individual’s therapeutic plan is revised accordingly [42]. Regarding the performance of a glucose meter, Cohen et al. revealed that the commercial version of glucometers such as CareSens and Accu-Chek are two of the most precise meters with a measurement error of 4.0 and 6.5%, respectively [43].

To overcome the limits of SMBG, CGM sensors have been developed that have been shown to improve the performance of diabetes management [15,16]. CGM systems are wearable medical devices that provide real-time measurements of subcutaneous glucose concentration almost continuously, e.g., every 1–5 min for several consecutive days. The CGM devices utilize a wire-based sensor, usually placed in the subcutaneous tissue, which measures the “raw” signal current via a glucose-oxidase electrochemical reaction. This electrical signal needs to be translated in real-time to glucose concentration through a calibration process [3]. A calibration procedure is required to collect one or more reference BG values from a portable SMBG device. Buckingham et al. investigated the relationship between calibration and CGM sensor accuracy [44]. The results showed that there was little improvement in sensor accuracy by including more than four calibrations each day. In addition, the sensor accuracy was most improved by obtaining calibration values when there was not a rapid rate of glucose change. Furthermore, Marvicsin et al. revealed that the Food and Drug Administration has approved CGM sensor for monitoring glucose trends and patterns, but it is not approved for the use of a single glucose reading at a specific moment in time, such as a glucometer reading, and therefore should not be used to calculate an insulin dose [45]. In addition, the authors suggested that the CGM sensor needed to be calibrated with actual BG readings 2–4 times per day.

It is expected that Internet of the Things (IoT)-based frameworks will rebuild the healthcare area with regard to public welfare, penetration, and cost efficiency [46,47]. An IoT-based monitoring system ensures the provision of benefits such as remote real-time health monitoring with fewer errors, diminished medical management costs, and enhanced patient experience and satisfaction. Recent advances in ICT along with novel biosensors that could provide a real-time monitoring condition of the patient offer a new perspective in diabetes management. Rodriguez-Rodriguez et al. suggested that a system architecture composed of sensors, a local gateway (smartphone), and a cloud system is the best candidate to improve diabetes management [17]. A glucose monitoring system collects the sensor data from sensors attached to the body. Moreover, as well as the low-power operation of the sensor node, communication between the sensor node and the smartphone requires the use of wireless technology. The best solution to fulfil such requirements is BLE [21], which is emerging as a key enabler of the IoT and allows the use of BLE-enabled devices such as smartphones as gateways responsible for collecting data from the sensor node via BLE [18]. Furthermore, the sensor data collected by the smartphone will be stored on a secure remote server and a web portal could be utilized to present the glucose level to the physician and patient, thereby helping to improve diabetes management [48].

BLE is an emerging wireless communication technology for short-range communication with a very low power consumption rate and can be operated for longer periods such as months and even years [19,20]. BLE have enormous impact on creating major changes in industrial sectors as well as create new businesses and opportunities. The previous studies have shown that the BLE-based wearable sensor device can provide the advantages in real-case implementations such as activity recognition [49], resident localization [50], indoor parking solution [51], multimedia streaming [52], and air quality monitoring [53]. Filippoupolitis et al. proposed an activity recognition system by utilizing a smart watch and BLE beacons [49]. Their results showed that by integrating with supervised machine-learning algorithms, the proposed activity recognition system was able to reach a classification accuracy ranging from 92 to 100%. Mokhtari et al. studied the performance of BLE technology for the purpose of resident localization and activity labelling [50]. The researchers investigated the impact of different advertising time intervals, power levels, and proposed an efficient and applicable algorithm to select optimal value settings for BLE sensors. A BLE-based navigation system for car searching in indoor parking garages has been proposed [51]. Their study utilized car-searching mobile app based on Android and used Bytereal HiBeacon devices. The experimental results showed that the proposed system has provided correct route guidance information to the car owner. Gentili et al. have utilized BLE for speech streaming service by proposing BlueVoice, an application that can demonstrates the feasibility of multimedia communication between sensor devices [52]. BlueVoice performance has been evaluated and showed high performance, and hence, it could be considered as a suitable solution for advanced applications in the IoT scenario. Suarez et al. proposed BLE-based monitoring system for environmental application and air quality detection [53]. The data obtained by temperature, humidity, and gas detection sensor were transmitted to the smartphone through a BLE communication. The machine learning based algorithms are used to predict 10 volatile organic compounds and showed that their accuracy is 88.33% and 92.22% for multilayer perceptron and radial-basis based neural networks, respectively. Additionally, it is important to investigate the impact of various critical parameters on BLE performance. The previous study revealed that the energy consumption, latency, piconet size, and throughput are mainly dependent on parameters such as *connInterval* and *connSlaveLatency* [21].

Zhang et al. [54] has provided an overview of the state-of-the-art of BLE technology for wearable sensor-based healthcare systems. They suggested that sensor technologies with a lower power communication, such as BLE devices, make it possible for wearable healthcare systems because of its light weight and is usable without location constrains. Additionally, Omre [55] noted that BLE is the first wireless communication technology for wearable healthcare devices that meets interoperability, electronic compatibility, secure data transmission, low-power operation, direct communication with cellular, and internet infrastructure. Rachim and Chung have proposed a low power transmission system to monitor heart activity through ECG signals by utilizing BLE as protocol for data transmission between sensors in the armband and smartphone [56]. The proposed system successfully detected the ECG signal from the arm and performed high accuracy as the error rate for health-rate calculation was less than 10% compared with a standard system. An android application was developed to present the sensor data in real time through BLE communication. Mora et al. proposed a distributed framework based on the IoT paradigm to monitor human biomedical signals in activities by utilizing a BLE-sensor device [57]. The main advantage and novelty of the proposed system was the flexibility in computing the health monitoring application by using resources from available devices inside the body area network of the user. The proposed model was validated by monitoring footballers’ heart rates during a football match. Also, the BLE technology has provided positive impact on diabetes self-management, thus it is expected to improve the health quality of diabetic patients. Arsand et al. presented an easy way to monitor BG, insulin injections, physical activity, and dietary information by utilizing smartphones and smartwatches [58]. The study utilized a BLE-based wearable device which transmitted the sensor data to the Diabetes Diary app on a smartphone through BLE. In addition, Cappon et al. investigated the features of current commercial version as well as research prototypes of CGM wearable sensor [59]. The study revealed that the current leading CGM, such as Dexcom G5, has utilized a wireless connection via BLE. Accordingly, the transfer data between a sensor and an app on the patient’s smartphone can be performed efficiently. The result showed that the present CGM hardware technologies and its software application could positively impact the daily life of patients.

Current technology allows the smartphone to be utilized as a gateway for medical devices as well as tools to monitor the BG level of a user. Morón et al. evaluated the performance of a smartphone as a medical gateway [60]. The study utilized a commercial android smartphone which acted as a gateway between a set of wireless medical sensors and a data server. The study also investigated the capabilities of current commercial smartphones for use as gateways in health monitoring applications. The performance of the model was presented based on the smartphone’s CPU, transmission rate, and power consumption. The conclusion of the study was that present commercial smartphones are sufficiently capable of combining their normal operation as phones or multimedia reproducers and simultaneously perform as medical monitors or gateways for healthcare. Furthermore, previous studies have demonstrated the capability of a smartphone to monitor the BG level of diabetic patients. Lee et al. proposed a system based on a Personal Digital Assistant (PDA) that can send information on the BG level, blood pressure, food consumption, exercise, etc. of a diabetic patient and manage the treatment by recommending and monitoring food consumption, physical activity, insulin dosage, etc. so that the patient can better manage his/her condition [61]. The proposed system was based on rules and the k-nearest neighbor (KNN) classifier algorithm to obtain the optimum treatment recommendation. Keith-Hynes et al. presented the Diabetes Assistant (DiAs) based on an android smartphone wirelessly connected to a CGM device and insulin pump for T1D patients [62]. The proposed model was able to monitor the real-time data from the CGM device and pump as well as transmit the data to a secure remote server. In addition, the proposed model could estimate the patient’s metabolic state and the risk of hypo- and hyperglycemia as well as adjust the insulin infusion rate. Finally, Garnweidner-Holme et al. proposed a smartphone application that automatically receives the sensor data from a glucometer [63]. The proposed application allowed the user to attain information regarding diet and physical activity. The authors suggested that involvement from relevant groups such as health professionals, patients, data security, and privacy experts and designers was necessary to develop such an application. Their results indicate that the proposed application could be utilized for gestational T1D patients to control their blood sugar levels as well as their healthcare treatment.

Currently, the number of BLE-based wearable sensors, smartphones, and other connected things are increasing significantly. Utilizing a BLE-sensor device allows a patient to self-monitor his/her BG in a comfortable way [58,59]. As the number of devices collecting patient and sensor data increases, the possibilities of using new types of applications that can handle the input of big amounts of health sensor data such as real-time data processing also increases. In addition, Rodriguez-Rodriguez revealed that sensor devices such as glucose and heart rate monitors will generate huge amount of data, and big data analysis can be introduced to solve this matter [17]. By utilizing real-time data processing, the enormous amount of data collected by many heterogeneous sources (sensor devices) can be handled and presented to the patient and medical team in an efficient way.

### 2.2. Real-Time Data Processing

Currently, the amount of data produced and shared by companies, governments, industrial sectors, and technical research is massively enlarging. Daily, the world generates around 2.5 quintillion bytes of data (e.g., 1 exabyte = 1 quintillion bytes, or 1 exabyte = 1 billion gigabytes), 90% of which is unstructured [64]. With the shocking amount of complex data that is produced by any device, anywhere, anytime, this is undeniably the era of big data. Due to the growing data trend, big data analytics can be utilized as operator for vendors’ competitive gain [65]. The volume of healthcare data including different and variable text types, sounds, and images is increasing day to day, so the storage and processing of these data is a necessary and challenging issue. There are several applications of big data analytics in the healthcare industry. Fahim et al. developed a system that can promote an active lifestyle and recommend valuable interventions to individuals [22]. The proposed model utilized a big data infrastructure, machine learning, and a cloud system for the massive amount of sensory data from smart devices and their fast retrieval for recommendations. Han et al. proposed a framework for a cardiovascular disease (CVD) prediction system based on lambda architecture to solve the problems associated with the real-time analyses of big data [23]. A variety of health data sources such as clinical, genomic, and lifestyle data could be utilized by the proposed framework to predict diseases and conditions. Finally, Huh investigated the capability of a big data analysis to be utilized for obesity healthcare management [24]. The study collected big data by applying machine learning and crawling methods to the unstructured citizen health data in Korea. The author suggested that the big data analysis using various keywords specific to a person would result in measures for personalized treatment and healthy activities, and thus improve the health quality of the individual.

Generally, relational DBs are used for storing health data, but they have limitation such as an inability to handle massive and unstructured sensor data. The NoSQL DB has arisen with the purpose of offering better solutions and features to handle massive amounts of data with high performance, sometimes near real-time [25]. The NoSQL architecture provides significant advantages in storage and query efficiency, thereby reducing the cost of data management. In the past few years, usage of the NoSQL DB has been widespread in different sectors because of its ability to deal with new requirements of applications. It presents a new storage architecture that fulfils high scalability, availability, and fast retrieval requirements for managing unstructured and partially structured data. Several open-source technologies can be used to handle the incoming large volume of data quickly, such as Apache Kafka and NoSQL MongoDB. Apache Kafka handles the incoming data, whereas MongoDB is utilized to store unstructured data. Apache Kafka is a tool that can be used as a publish-subscribe messaging system in real-time [66]. MongoDB is an open source NoSQL DB that saves data in documents instead of fixed tables needed by relational database management systems [67].

Several studies have been conducted regarding the performance of Apache Kafka as a real-time messaging system. Kreps et al. introduced Kafka, a distributed messaging system for collecting and delivering high volumes of log data with low latency [68]. The results of their experiments showed that Kafka had superior performance when compared to other messaging systems. A recent study utilized Apache Kafka for stream processing. Fernandez-Rodriguez et al. proposed real-time data streaming for vehicles in a smart city [69]. Their results revealed that Apache Kafka achieved a greater scalability, faster response, and reduced cost compared to traditional systems. Syafrudin et al. proposed an open source-based real-time data processing architecture consisting of several open sources technologies, including Apache Kafka [70]. Their results show that the proposed system was capable of processing a massive amount of sensor data efficiently when the amount of it and the number of devices increased.

The use of sensors is increasing and has led to an increased demand for sensor data storage platforms. NoSQL DBs have gained momentum in the last couple of years because of the growing scalability and availability requirements. A previous study compared the performance of one open source SQL DB (PostgreSQL) and two open source NoSQL DBs (Cassandra and MongoDB) [25]. The results suggest that MongoDB is the best choice for a small- or medium-sized non-critical sensor application, especially when the write performance is important. Schulz compared the performance of common relational DBs with document-oriented NoSQL DBs [26]. The results of the study revealed that the NoSQL architecture outperformed the traditional relational models in terms of data storage speed, indexing, and query retrieval on nearly every operation. Pereira et al. presented a study on NoSQL DBs and evaluated the performance of three different NoSQL DBs such as Couchbase, MongoDB, and RethinkDB [27]. Tests were performed in two different scenarios—single thread and multiple threads—and the results revealed that for retrieving multiple documents and inserting documents with multiple threads, the MongoDB showed a better performance. Finally, Hu evaluated the performance of six popular DBs (Rasdaman, SciDB, Spark, ClimateSpark, Hive, and MongoDB) for handling multi-dimensional array-based geospatial raster datasets [28]. The system performances were presented based on the system design architecture and practical use experience. Their results showed that SciDB and MongoDB had better query performances compared to other databases. However, their performances were limited when handling large data volumes due to their heavy consumption of memory.

Regarding the application of MongoDB in the healthcare industry, several studies have been conducted. Goli-Malekabadi proposed a model based on NoSQL DBs for the storage of health data [29]. The presented model was implemented in a Cloud environment to assess the distribution properties. The efficiency of the model was evaluated based on query time, data preparation, flexibility, and extensibility parameters. The results show that the presented model was more effective than relational DBs, especially for handling health data. Nkenyereye proposed a Remote Healthcare Monitoring application for patients residing in an isolated village [30]. The study utilized Node.js, a cross-platform runtime environment featuring technologies for handling concurrency issues. The experimental results of the study showed that server-side JavaScript with Node.js and MongoDB was 40% faster than the other methods tested.

Integration of Apache Kafka and MongoDB can be utilized for real-time data processing for handling healthcare sensor data. Previous studies have shown that both technologies can be utilized for real-time data processing so that an incoming large volume of streaming data can be quickly handled, stored, and presented in real-time [69,70]. Thus, in our study, Apache Kafka and NoSQL MongoDB were used for real-time data processing to monitor the vital signs data from diabetic patients and presented in real time. In addition, by integrating real-time data processing with machine-learning algorithms, we expected to help patients to better self-manage diabetes.

### 2.3. Machine Learning–Based Algorithms for Diabetes

T2D is a progressive condition in which the body becomes resistant to the normal effects of insulin and/or gradually loses the capacity to produce enough insulin in the pancreas. Most of the people with T2D display no symptoms, thus screening to detect pre-diabetes and diabetes should be considered in individuals ≥45 years of age, particularly in those with a BMI ≥ 25 kg/m^2^ [2]. That is probably because people tend to exercise less, lose muscle mass, and gain weight as they age. Thus, an automatic prediction model that warns people of the chances of developing diabetes in the future is necessary and would enable them to take preventive action. Machine-learning algorithms can be utilized to classify diabetes based on the current condition of a patient. Supervised learning classification models are popular machine-learning methods used with the aim of constructing a classification model from a training set to be used to predict class labels for the given data by creating datasets containing DB tuples and their associated class labels [71]. Generally, supervised learning classification models must perform the following steps: (i) data collection; (ii) feature extraction; (iii) selection of a machine-learning algorithm; (iv) model construction using the selected algorithm; and (v) evaluation of the algorithm accuracy.

Several studies have been conducted regarding diabetes classification that utilized clinical datasets such as the PIMA Indian dataset. The dataset consists of 768 patients, of which 268 patients are diabetic and 500 patients are normal. Maniruzzaman et al. proposed a Gaussian process (GP)-based classification technique using three kernels, namely linear, polynomial, and radial basis [31]. The proposed model was compared to existing techniques such as linear discriminant analysis, quadratic discriminant analysis, and Naïve Bayes (NB). The result showed that the performance of a GP-based model is higher compared to other methods. Cheruku et al. proposed SM-RuleMiner for diabetes classification [32]. The proposed rule-miner is compared against rule-based algorithms such as ID3, C4.5, and CART along with several meta-heuristic-based rule mining algorithms. The results of the study showed that the proposed SM-RuleMiner outperformed the other algorithms in terms of average classification accuracy and average sensitivity. In addition, Wu et al. proposed a novel model based on data mining techniques for predicting T2D [33]. It was comprised of two parts: an improved K-means algorithm and the logistic regression algorithm. The improved K-means algorithm was utilized to remove incorrectly clustered data, after which the logistic regression algorithm was used to classify the remaining data. The results revealed that the proposed model showed a higher prediction accuracy compared to previous studies.

Providing a diabetes detection system by using input data containing only medical information without advanced medical equipment could help a number of people discover the risk of diabetes at an early stage. Several studies have demonstrated such a system that could possibly have a huge positive impact on a lot of people’s lives. Meng et al. compared the performance of logistic regression, artificial neural networks (ANNs), and decision tree models for predicting diabetes or prediabetes using common risk factors [34]. Participants came from two communities in Guangzhou, China, of which 735 patients were confirmed as having diabetes or prediabetes and 752 were normal controls. The study utilized a standard questionnaire to obtain information related to demographic characteristics, family diabetes history, anthropometric measurements, and lifestyle risk factors. The classification model utilized 12 input variables and one output variable from the questionnaire information. Their results showed that decision tree model C5.0 provided the best classification accuracy compared to the other ones. Perveen et al. proposed a model to classify patients with diabetes using diabetes risk factors [35]. A dataset was obtained from the CPCSSN DB containing 667,907 records for a period ranging from 2003 through 2013. Each record contained several features including important risk factors such as vital signs, diagnosis, and demographics that were used to predict diabetes. Their experimental result showed that the Adaboost ensemble method performed better than bagging and the standalone J48 decision tree. Finally, Nai-arun et al. compared classification models such as Random Forest, Decision Tree, ANN, Logistic Regression, and NB for diabetes risk classification [36]. The dataset was collected from 30,122 people from 26 Primary Care Units in Sawanpracharak Regional Hospital, Thailand, during 2012–2013. Medical information such as BMI, age, weight, height, blood pressure, a history of diabetes in the family, a history of hypertension in the family, gender, and alcohol and smoking patterns were utilized as input features while the class was either the diabetes risk or normal. The results of the study show that Random Forest provided the best performance compared to the other algorithms.

T1D is a chronic disease requiring patients to know their BG values in order to ensure their BG levels as close to the normal range as possible. Several studies have demonstrated BG prediction, which can help patients to obtain future predictions of their BG level so that preventive alerts can be generated before critical hypo/hyperglycemic events occur. Sparacino et al. compared the predictive accuracy of a first-order autoregressive model with a first-order polynomial model [37]. For each model, the inputs were past BG levels. These approaches assessed data from T1D patients recorded every 3 min using a CGM device. The results demonstrated that models could predict hypo/hyperglycemic events more than 20–25 min ahead in time. Plis et al. described a solution that uses a generic physiological model of BG to be used by Support Vector Regression (SVR) to predict the BG level [38]. Their study showed that the proposed model outperformed diabetes experts at predicting BG levels and could be used to anticipate almost a quarter of the hypoglycemic events 30 min in advance. Ahmed et al. proposed BG prediction based on a Neural Network and a Kalman filter and utilized the BG data from a CGM device [39]. Their study showed that the proposed model could predict the BG level accurately for up to 2 h. The study also revealed that external factors such as body weight, exercise pattern, and lifestyle might have influence how well the model could predict the BG level. Finally, Hamdi et al. proposed weighted SVR based on the differential evolution algorithm [40]. The proposed method is validated using real CGM data of 12 patients. The obtained root-mean-square error (RMSE) averages were 9.44, 10.78, 11.82, and 12.95 mg/dL for prediction horizons of 15, 30, 45, and 60 min, respectively.

The current scholar literatures still lacks the studies focusing on system integration of BLE-based sensor device, smartphone, real-time data processing as well as machine learning-based methods to predict the diabetes, and BG levels. Therefore, it is necessary to integrate the aforementioned technologies to improve the diabetes management. The application of machine-learning algorithms can help individuals to predict the possibility of diabetes along with future BG levels. The proposed model is expected to help individuals in real-time to monitor their vital signs data from BLE-based sensor and smartphone. Additionally, it helps individuals to discover the risk of diabetes at an early stage as well as help patients to obtain future predictions of their BG levels. In this way, an individual can avoid the worst conditions in the future.

## 3. Methodology

### 3.1. System Design

In this study, a personalized healthcare monitoring system was developed to help patients to better self-manage their chronic condition. The proposed system records various health-related activities of the users. Moreover, it can be considered as a healthcare information platform that achieves interaction between patients, medical institutions, and medical devices over a wireless network. The main idea behind the system is to collect users’ vital signs data using sensors and then transfer the data over a wireless network to a remote service platform. After this, with the help of machine-learning methods, it can help users to review their ongoing health patterns and predict future changes in health status.

**Figure 1:**
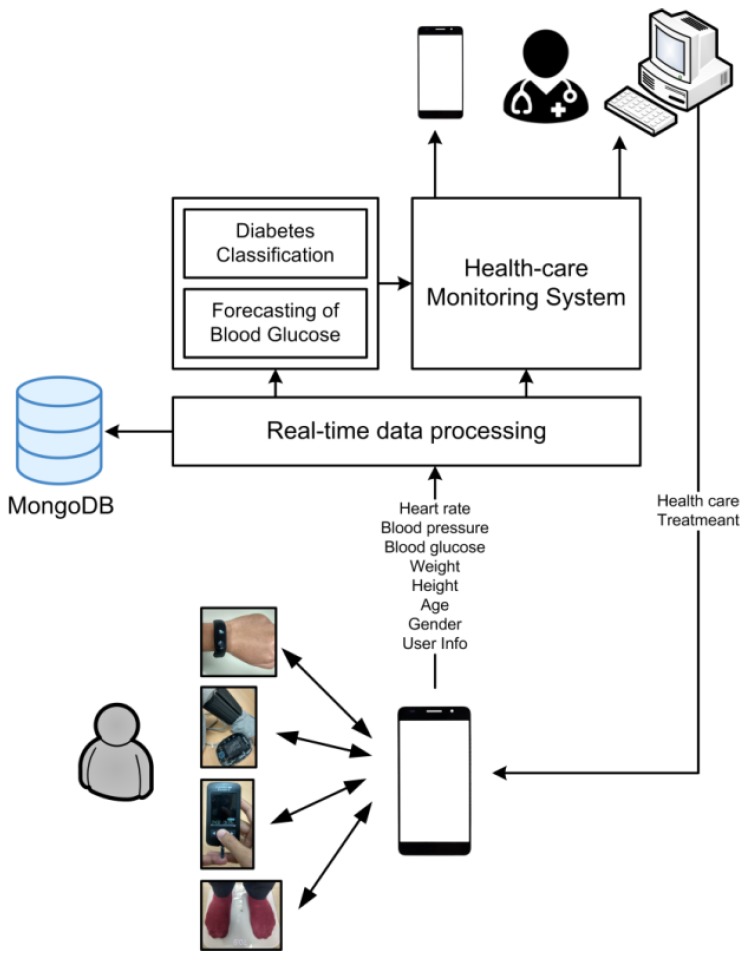
The architecture of the personalized healthcare monitoring system for diabetic patients.

As can be seen in Figure 1, the BLE-based sensors collect the user’s vital signs data and then transfer the data via Bluetooth to the smartphone. A BLE-based device such as a smart band, a blood pressure monitor, weight scales, and a glucometer sensor are utilized to collect user data such as heart rate, blood pressure, weight, and BG level. A prototype android app was developed to receive the user’s vital signs data from the sensors as well as user personal input (gender, height, age, and other information). The sensor and personal data are transmitted wirelessly to a secure remote server where the real-time data processing is installed, which allows the system to handle the huge amount of sensor data quickly before it is stored in the MongoDB. Two machine learning–based solutions for diabetes classification and forecasting of BG are utilized to analyze and predict future changes in health status given current user personal sensor data. MLP is utilized to predict the presence or possibility of diabetes in the future, while LSTM is utilized to predict the future level of BG. The results of the analysis are then presented to the medical team via a web-based healthcare monitoring system. The results are combined with standard medical care from a doctor and the final personal healthcare treatment is delivered to the patient.

**Figure 2:**
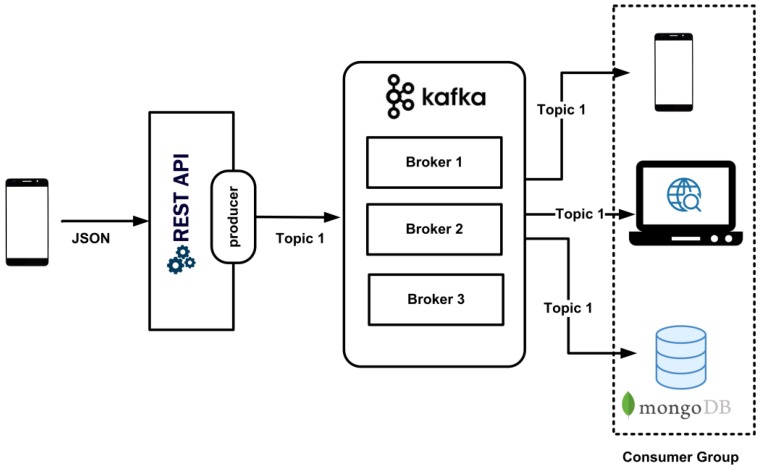
System design of the real-time data processing.

The proposed real-time data processing utilizes Apache Kafka as a streaming platform which provides low-latency, high-throughput, fault-tolerant publishing, and subscribed pipelines and is able to process streams of events. Figure 2 shows the system design of the real-time data processing for the healthcare monitoring system. The vital signs data from the patient is formatted in JSON and wirelessly transmitted using the developed android app by employing HTTP to the REST API in the secure remote server. The Node.js web application is utilized as the REST API to receive the sensor data and act as a “producer.” The “producer” publishes streams of records to one or more Kafka “topics” distributed across one or more cluster nodes/servers called the “broker”. Other processes called “consumers” allow the application to subscribe to the “topics” and process the stream of records. The “consumers” include the MongoDB, the web-based monitoring system, and the android app developed to store the sensor data and present health-related activities in real-time to both sets of users: the patients and the medical team.

**Figure 3:**
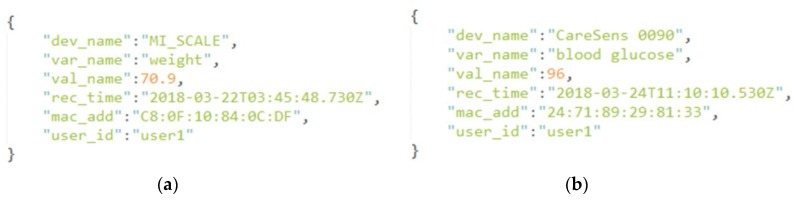
An example of user weight (**a**) and user blood glucose (BG) (**b**) sensor data in the NoSQL MongoDB.

The amount of generated sensor data from Kafka is large and is continuously generated. In this project, the NoSQL MongoDB was considered as the DB to store the sensor data from the patients. Figure 3a shows an example of a sensor document generated from the weight scales device while Figure 3b presents a sensor document of the BG level of a patient. A sensor document consists of a sensor device name and its unique mac address, a variable name and its value, the recorded time, and the unique ID of the patient.

### 3.2. System Implementation

**Figure 4:**
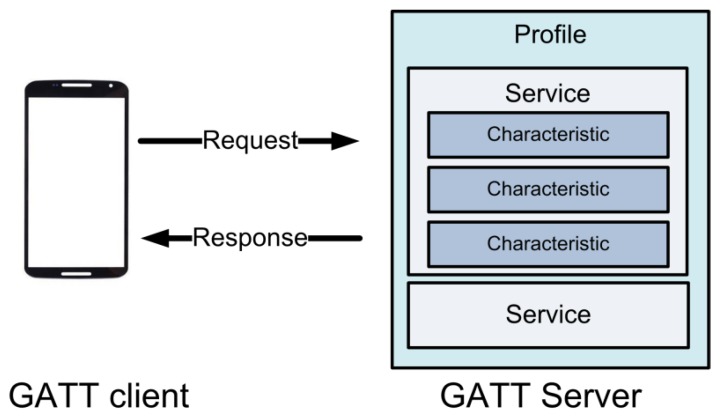
A diagram of the Generic Attributes (GATT) server.

With today’s technology, it is possible to implement a wireless solution as a replacement for wires or cables that can exchange information. BLE can be considered as the best choice for sending sensor data continuously while maintaining low power consumption. In this study, BLE-based wearable sensors such as blood glucometers, smart bands, blood pressure monitors, and weight scales were utilized to monitor the vital signs data of the users. Communication between the BLE-based sensors and a smartphone was defined using Generic Attributes (GATT) [72]. A BLE peripheral (sensor device) can only be connected to one central device (e.g., smartphone) at a time, but the central device can be connected to multiple peripherals. GATT transactions in BLE are based on high-level nested objects called Profiles, Services, and Characteristics, as shown in Figure 4. The lowest level concept in GATT transactions is the Characteristic, which encapsulates a single sensor data. In order to enable communication with the sensor device, the smartphone must scan nearby devices and decide which device the central device should connect to. Once the connection is established, the Service and Characteristics can be discovered, after which the sensor data can be transmitted to the smartphone.

**Figure 5:**
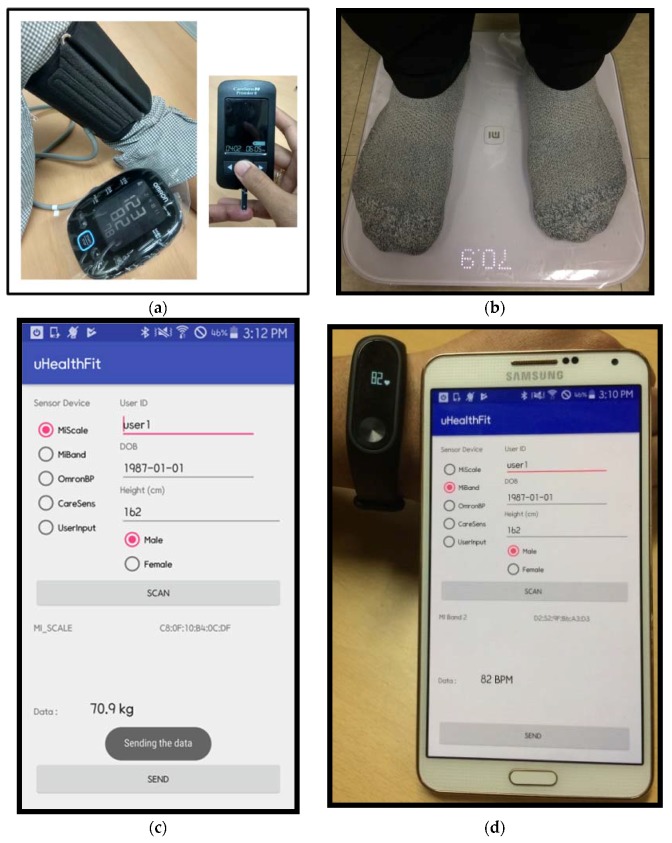
(**a**) The blood pressure and blood glucometer devices; (**b**) weight measurement by a user; (**c**) weight data presented in real-time by the android app; and (**d**) the heart rate sensor in operation.

In this project, commercial versions of BLE-based wearable sensors: Mi Band 2, Mi Smart Scale, Caresens, and Omron were utilized for monitoring the heart rate, weight, BG, and blood pressure of the user, respectively. We developed a prototype of an android app called uHealthFit based on the GATT concept, which made it possible to retrieve the vital signs data on blood pressure, BG, weight, and heart rate from the multiple BLE-based sensor devices to the smartphone. The smartphone continuously received sensor data from the sensor devices and presented it in the android app interface. The smartphone acted as an information gateway by gathering information from the BLE-based sensors, retransmitting it to the secure remote server wirelessly, and storing it in the MongoDB. Figure 5a shows the blood pressure monitor and blood glucometer in operation, while Figure 5b shows the weight measurement process for a user. First, the uHealthFit prototype was connected to one of the sensor devices (by selecting one of the radio buttons on the screen) and the “scan” button was pressed. Once a connection had been established, the sensor device name and its mac address were presented on the screen. Figure 5c shows user weight data being presented in real-time by the uHealthFit app while Figure 5d exhibits heart rate data being retrieved from a Mi Band 2 and presented on the app screen. By selecting the different options of sensor device, the sensor data from each sensor device could be gathered by the app. In addition, by selecting the “User Input” radio button, the user was able to provide other personal data manually, such as user id, date of birth, height, and gender information. Once the sensor data and personal data had been presented in the app, the user stored the health-related activities data to the secure remote server by pressing the “send” button.

### 3.3. Diabetes Classification and BG Prediction.

The main idea behind the diabetes classification in this study is for the early prediction of diabetes given a user’s vital signs data from the BLE-based sensors. Early disease prediction can lead to treating the patients before the condition becomes critical. Datasets from the PIMA Indians Diabetes Database at the National Institute of Diabetes and Digestive and Kidney Diseases was utilized in this study [73]. These datasets include records of 768 patients out of which 500 tested negative (normal) while 268 of them tested positive. The objective of using the dataset was to predict diagnostically whether a patient had developed diabetes within five years in PIMA Indian women. There was input for eight variables in the dataset: the number of pregnancies, 2 h glucose tolerance, diastolic blood pressure, triceps skinfold, 2 h serum insulin, body mass index, diabetes pedigree function, and age. In this study, only four attributes were utilized for training the classification model. The attributes selection was based on the similarity of the vital signs data from our BLE-based sensor devices: 2 h glucose tolerance, diastolic blood pressure, body mass index, and age. The diastolic blood pressure value could have been generated from a blood pressure sensor device, while the 2 h glucose tolerance value was similar to that attained from the blood glucometer. In addition, our developed android app received the personal input data from the users such as date of birth and height. Those inputs were combined with the weight scales sensor data to present the age and body mass index (BMI).

**Figure 6:**
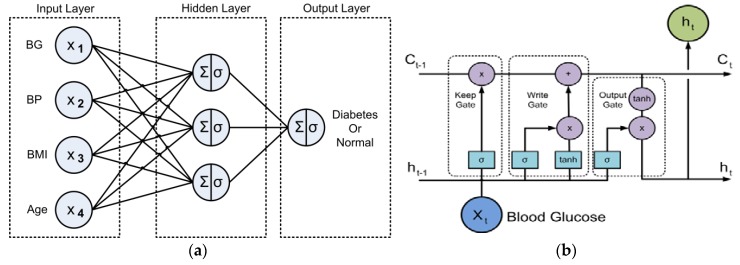
(**a**) The Multilayer Perceptron (MLP) architecture for diabetes classification and (**b**) BG prediction based on Long Short-Term Memory (LSTM).

Figure 6a shows the MLP architecture for diabetes classification. MLP is a network consisting of a set of source nodes forming an input layer, one or more hidden layers, and an output layer of the nodes. The algorithm utilized backpropagation to update a set of weights for predicting the class labels [71,74,75]. In this study, the 2 h glucose tolerance (Blood Glucose/BG), diastolic blood pressure (BP), Body Mass Index (BMI), and Age input nodes were presented to the input layer, after which the input data was fed to the network’s input layer. Next, the net input to the unit was computed by multiplying each input connected to the unit and its corresponding weight, and this was summed. Each unit in the hidden layer took its net input and then applied an activation function (sigmoid) to it, thus it could transfer the large input onto a smaller range of 0 to 1. This computation was applied for each hidden layer and output layer, which enables the network’s predictions. MLP utilized backpropagation by comparing the network’s prediction for each tuple with the actual target class value. For each training tuple, the weights were modified to minimize the mean-squared error between the network’s prediction and the actual target value. These modifications were made in the “backward” direction, from the output layer to the first hidden layer. This process was iterated multiple times, thus in the end the network with its optimal weights was able to present predictions using the testing data.

Furthermore, the BG prediction based on the user sensor data was also presented in this study. Two BG datasets were utilized. The first diabetes dataset was provided by Michael Kahn, MD, PhD, Washington University [76]. The dataset consisted of the records of diabetic patients for different time intervals: breakfast, lunch, dinner, and bedtime. The 70 diabetic patients were monitored and data such as insulin dose, BG measurement, meal ingestion, and exercise activity were recorded in each different time interval. In this study, we only utilized a single input (the BG level) for the training model. Other BG dataset was provided by Andy Choens [77]. This dataset comprised the BG levels of a single T1D patient recorded every 5 min. The dataset consisted of data from a DexCom G4 CGM device.

LSTM was utilized in this study due to its ability to transmit important information many time steps into the future [75,78]. Figure 6b shows the architecture of BG prediction based on LSTM. One of the core components of LSTM is *memory cell* (C), which hold critical information that is has learned over time. At each time step, the LSTM unit modifies *memory cell* based on three different phases: *keep gate*, *write gate*, and *output gate. Keep gate* determines how much of the previous memory to keep. The calculation is carried out by concatenating the input of this time step and the LSTM unit’s output from the previous time step and applying a sigmoid layer. The sigmoid layer output is then multiplied with the *memory cell* from previous state. If the output is 1, it means that *memory cell* is still relevant and ought to be kept. Output 0 means that *memory cell* is no longer relevant and needs to be erased. The next part is known as *write gate*, which determines what kind of information will be written into *memory cell.* This has two parts: a sigmoid layer to decide which values to update and a tanh layer to define the information written into *memory cell*. In the next step, the sigmoid and tanh layer outputs are combined to create a new *memory cell*. Finally, *output gate* is presented by the LSTM unit to provide the output. *Memory cell* is put through tanh gate, which multiplies it by the output of the sigmoid gate so that the final output can be produced. In this study, we utilized the LSTM model to predict the future BG level (h_t_) based on the current BG level (X_t_) of the user.

## 4. Result and Discussion

### 4.1. The Healthcare Monitoring System

**Figure 7:**
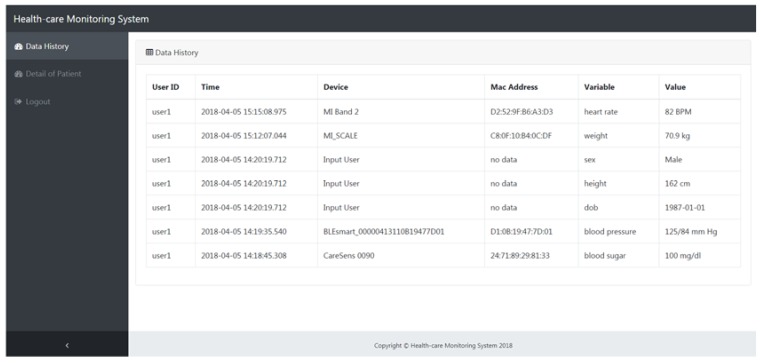
The web-based personalized healthcare monitoring system.

In this study, a web-based personalized healthcare monitoring system to receive and present the users’ vital signs data in real-time was developed. The proposed system can be utilized by patients and medical institutions to help patients to better self-manage their chronic condition. The sensor data sent by BLE-based sensor devices were processed by Apache Kafka in real-time and stored in NoSQL MongoDB. Apache Kafka also sent the topic (sensor data) directly to the web-based personalized healthcare monitoring system developed using NodeJS for presentation to the users in real-time. Figure 7 shows a prototype of the web-based healthcare monitoring system installed on the server side and easily accessible through a personal device (computer, laptop, or smartphone) by clients with an internet connection. The sensor data such as heart rate, weight, blood pressure, and BG combined with personal data such as gender, date of birth, and height were presented in the system for each user. For each data record, the device name (BLE-based sensor device), mac address of the sensor device, and the variable and its value was collected based on the recorded time. By monitoring the users’ vital signs data continuously, the complete history of a user’s vital signs can be collected and ready for further analysis using machine learning-based algorithms.

The proposed personalized healthcare monitoring system should be scalable to accommodate the growing volume of sensor data and sensor devices without suffering a noticeable performance loss. Our proposed personalized healthcare monitoring system consists of two parts: the real-time data processing and the BLE-based sensor application, and so a performance evaluation is presented for each part. The system latency, throughput, and concurrency are presented for the evaluation of the real-time data processing [25,27]. We defined response time as the time needed for the real-time data processing to store the sensor data in MongoDB, which reveals the system latency. The testing was combined with different numbers of clients (concurrency), thus the response time could be presented for the sensor data of each different number of clients. The throughput per second was used for the throughput testing and defined as the total amount of sensor data stored in the DB per second. In this study, a simulation was used as a client computer running a Java Program that generated sensor data connected to a single server machine running Apache Kafka and the NoSQL DB. The Java program used threads to simulate multiple clients. Both types of performance testing (the latency and throughput) were performed based on the number of sensor documents. A CPU with 4.20 GHz × 8 cores and 16 GB RAM was used as the server while a CPU with 2.53 GHz × 8 cores and 16 GB RAM was used as the client.

**Figure 8:**
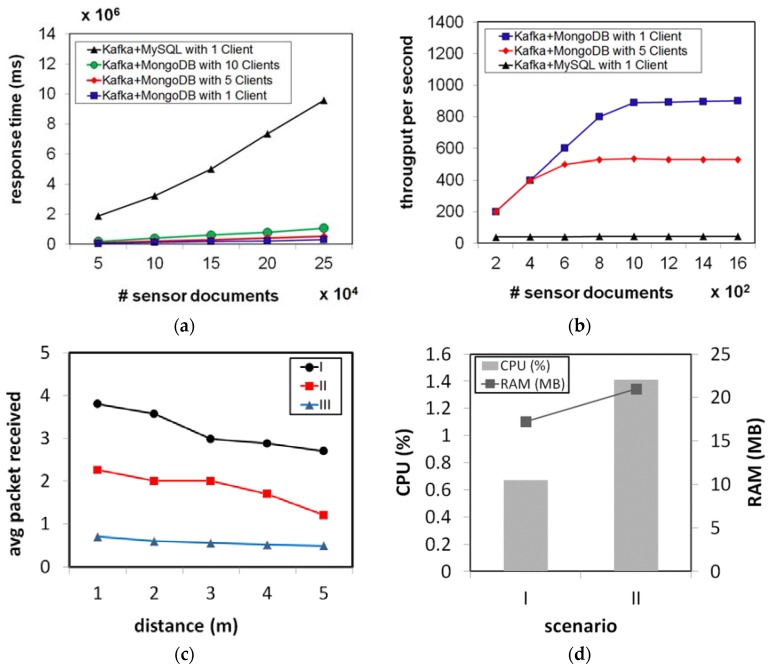
Performance testing of the proposed healthcare system: (**a**) write testing; (**b**) throughput testing; (**c**) the average data packets received; and (**d**) CPU and memory usage.

Figure 8a displays that as the amount of sensor data sent to the server increased, the response time also increased. This result reveals that by integrating Apache Kafka and MongoDB, the proposed real-time data processing performed better than the combination of Kafka and MySQL. The NoSQL MongoDB needed less time to store the sensor documents in storage than MySQL did. The number of clients also affected the response time, since more time was required for the proposed real-time data processing to store sensor data sent by a larger number of clients at the same time. However, for the multiple clients, the proposed real-time data processing still outperformed the traditional model as it had a lower response time for 10 clients to access the proposed model compared to MySQL accessed by a single client. In addition, Figure 8b shows the system throughput and reveals that the proposed system performed better because it could store sensor data sent by a single client 20 times higher than MySQL could in 1 s. Furthermore, the proposed model showed a high performance for multiple clients as the proposed model could store sensor data sent by five clients 11 times higher compared to MySQL did in 1 s for a single client.

A BLE-based sensor application consists of a BLE-based sensor device and the *uHealthFit* android app to retrieve sensor data and send them to a secure remote server. It is important to analyze the BLE-based sensor performance under various conditions. Two performance metrics, packet delivery and CPU, as well as memory usage were utilized in this study. Considering the data transfer from the BLE-based sensor (peripheral) to the smartphone (central device), the packet delivery performance was presented as the average number of data packets received by the smartphone at different distances during a 1 min period. The second performance metric was the average CPU and memory usage of the *uHealthFit* android app during 1 min of operation under various scenarios. In a previous study, the smartphone capability as a gateway was investigated based on CPU usage in different scenarios [60]. In our experiment, the Mi Smart Scale was utilized as the peripheral and a Samsung Galaxy Note 3 with android OS V5, 3 GB RAM, and a Li-Ion 3200 mAh battery was used as the central device.

Figure 8c shows the average packet received by the smartphone within 1 min of operation under different scenarios. In Scenario I, the smartphone receives the sensor data from a BLE-based sensor which was currently being operated by the user. In Scenario II, the smartphone connected to multiple peripherals via Bluetooth as the user operated a BLE-based sensor and a Bluetooth headset (listening to a song) at the same time. Scenario III had the smartphone connected to a BLE-based sensor in the idle position (advertising mode). The indoor environment testing was performed and different distances between the peripheral and the central device were tested, after which the results were collected and analyzed. The results show that when the BLE-based sensor was operated by the user (Scenarios I and II), the average data packet received by the android app was higher compared to the BLE-based sensor in the idle position (Scenario III). When the BLE-based sensor is not operated by the user, the peripheral sends out a bit of information that any device in the area can pick up (advertising mode). The number of connected device also affected the system performance as the results show that when the user operated the BLE-sensor device and listened to music at the same time (Scenario II), the average data packet received was lower than Scenario I. For all the scenarios, as the distance increased, the average data packet received decreased. Hence, increasing the distance between the peripheral and the device reduces the chances of receiving a transmission within acceptable RSSI levels.

Figure 8d shows the CPU and memory usage of the *uHealthFit* android app in two different scenarios. Scenario I was where the android app only received sensor data from a BLE-based sensor device and Scenario II was where the app received sensor data from the BLE-sensor device and sent it to the secure remote server simultaneously. Communication with the server side was implemented via a Wi-Fi connection. In particular, a Wi-Fi access point located within 1–3 m in line-of-sight of the smartphone was utilized for the experiment. Both scenarios were evaluated for 1 min of operation and the average CPU and memory usage were collected and analyzed. The results showed that the CPU and memory usage of the android app were affected by the data transmission to the secure remote server. When the app was receiving sensor data from the BLE-device alone, it required less CPU and memory usage than it did when the data were sent to the server at the same time as the app was running. Regarding the computational cost of the android app, it should be noted that it used less than 1.5% CPU and 22 MB during both scenarios.

### 4.2. Diabetes Classification and BG Prediction

The real-time data processing receives the sensor data from the device and stores it to the NoSQL MongoDB. Next, the sensor data is analyzed based on machine-learning algorithms to predict future changes in health status given the current data of the user. Two functionalities are presented in this study: diabetes classification and BG prediction. The detailed results of each prediction model are presented one after the other.

The diabetes classification utilized PIMA dataset [73] was used for the training and testing of the classification models. In this study, we only utilized four features for training and testing the models. The attributes were selected based on their similarity with our sensor devices’ output: 2 h glucose tolerance, diastolic blood pressure, body mass index, and age. The output attribute was whether a patient would have diabetes within five years. The performance metrics of the classification model were measured based on precision, recall, and accuracy and are presented in Table 1. TP and TN indicate the numbers of diabetes and normal patients that were correctly classified, respectively, while FN and FP indicate the numbers of normal and diabetes patients that were incorrectly classified, respectively. 10-fold cross-validation was used to train and test the dataset for the entire classification model.

Table 2 summarizes the classification model performance. Classification algorithms Random Forest, NB, Support Vector Machine (SVM), and Logistic Regression, were compared with MLP to learn and predict the PIMA dataset. The results show that MLP achieved the highest accuracy (77.083%) compared to 73.046%, 76.6927%, 76.562%, and 76.0417% for Random Forest, Naïve Bayes, SVM, and Logistic Regression, respectively. These results show that for a small number of features (2 h glucose tolerance, diastolic blood pressure, body mass index, and age), the MLP algorithm achieved the highest accuracy of prediction compared to other models. Thus, it is expected that the proposed MLP model could be utilized for diabetes detection for our proposed system given the input data from BLE-based sensor devices (e.g., blood glucometer, blood pressure monitor, and weight scales) and personal user input (e.g., height and date of birth).

In addition, BG prediction based on LSTM is presented in this study. Two datasets were utilized: dataset 1 from the UCI repository [76] and Andy Choens’ dataset [77] as dataset 2. For dataset 1, 70 diabetes patients were monitored and the data such as insulin dose, BG measurement, meal ingestion, exercise activity were recorded at each time interval. In the present study, we analyzed the daily data of BG measurements before breakfast only (05.00–08.00) and removed the other features. By using this scenario, the LSTM model was expected to predict the BG level for the next day. The size of the dataset was another limitation in this study, thus we selected the single patient data (patient ID 30) with the largest number of records (148) for training and testing LSTM. The dataset contained the BG level history recorded for patient ID 30 before his/her breakfast every day from 18 December 1990 until 18 May 1991. Furthermore, dataset 2 contained the BG level records of a single diabetes patient taken every 5 min. The collection method for the second dataset was different from the first one in that a different type of sensor (a CGM device) was utilized. The CGM device allows the user to record the BG level every 5 min, thus it can collect a large amount of data. After the cleaning stage, 26,167 records were available for the reading period between 20 February 2015 15:34 and 16 September, 21:33. Both datasets were split into two parts: approximately 80% for training, and the remainder for testing.

The performance metrics of the forecasting model were measured based on the correlation coefficient (*r*) and RMSE. The correlation coefficient is widely used as a measure of the strength of linear dependence between two variables (the actual and predicted values), while RMSE gives an indication of the overall accuracy of the approximation. The correlation coefficient has a value between +1 and −1, where 1 is a total positive linear correlation, 0 is no linear correlation, and −1 is a total negative linear correlation. RMSE can be utilized to measure how large an error there is between the predicted and actual values. The detailed formula of r and RMSE for a dataset of size *n*, actual value *y*, and predicted value $\hat{y}$ is presented in Table 3.

Table 4 contains the performance of forecasting models based on RMSE and *r*. The forecasting models linear regression and moving average were compared with LSTM to predict the BG based on the two different datasets. The results show that LSTM achieved the highest performance compared to the others for both datasets. For dataset 1, the correlation coefficient of LSTM was 0.647 compared to −0.019 and −0.183 for linear regression and moving average, respectively. It should be noticed that LSTM performed better when the size of dataset increased, as can be seen in the results for dataset 2. The correlation coefficient of LSTM was 0.999 compared to −0.071 and 0.710 for linear regression and moving average respectively. In addition, LSTM showed the highest performance as it generated the lowest RMSE compared to other methods: 25.621 and 2.285 for datasets 1 and 2, respectively. Integrating LSTM into the proposed personalized healthcare monitoring system ensures a high accuracy of BG prediction given the current input data from BLE-based sensor devices (e.g., blood glucometer, CGM device).

**Figure 9:**
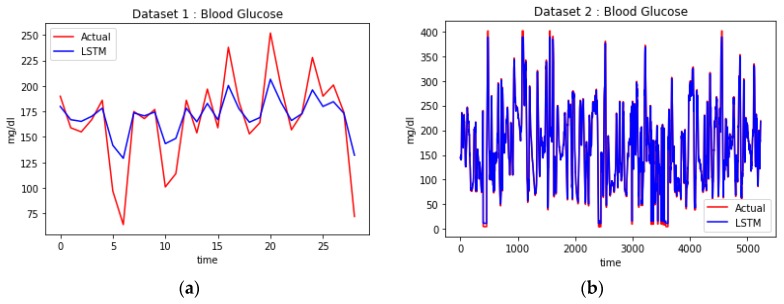
BG prediction based on LSTM for first (**a**) and second dataset (**b**).

The LSTM network is a type of recurrent neural network (RNN) capable of learning over long sequences. Compared to RNNs, an LSTM specifically implements *memory cells* with the capability to store memory for longer periods of time. LSTMs have a *keep gate* that controls whether the input at each time step is added to the *memory cell* or not. These capabilities affected the results of our experiment as LSTM showed the highest performance compared to the other methods. Two types of BG sensor, namely a glucometer and a CGM device, were utilized to gather the glucose sensor data, which was then split into training (80%) and testing (20%) sets. The training data at each time step was learned by LSTM to generate the learning model used to predict the testing data. As can be seen in Figure 9a, the LSTM model closely predicted the BG testing data, while in Figure 9b showed that, as the dataset size increased, LSTM more accurately predicted the BG testing data.

### 4.3. The Implications for Diabetes Management

The result of this study could be applied to any end-user application, such as the proposed android-based one. The app gathers the vital signs data from BLE-based sensor devices and stores them in a secure remote server. Two types of user are expected to utilize the android-based app: normal (currently not diagnosed as diabetic) and diabetic. The first usage is for normal patients who could utilize our BLE-based sensor device and healthcare monitoring system to check their current health status. Based on an American Diabetes Association (ADA) report [9], a person who is diagnosed with diabetes when the 2-h plasma glucose or random plasma glucose is at least 200 mg/dL or the fasting plasma glucose level ≥ 126 mg/dL. For a normal individual, the 2-h plasma glucose is expected to be lower than 140 mg/dL. By utilizing a personalized healthcare monitoring system, a user can check and monitor his/her current health status concerning BG, blood pressure, weight, and heart rate. In addition, once the dataset from a real-case implementation is collected, the diabetes classification based on MLP can be utilized by the user to predict any health changes in the future. The input from BLE-based sensor devices (e.g., BG, blood pressure, weight scales) and personal data (e.g., height and date of birth) can be used by the proposed MLP model to predict whether the normal patient will get diabetes in the future.

The second usage of the proposed application is for diabetes patients as a personal utility to control/self-manage their chronic condition. Diabetic patients must control their level of BG by drug administration, a healthy diet, and physical activity in order to improve glycemic control and health quality. Diabetic patients are expected to control their BG level in the safe range 70–180 mg/dL. Hyperglycemia (BG > 180 mg/dL) can result in long-term complications, e.g., retinopathy, nephropathy, and CVD, while hypoglycaemia (BG < 70 mg/dL) can produce short-term adverse conditions that can cause coma or even death [9]. By utilizing a personalized healthcare monitoring system, the complete history of the user’s vital signs data can be stored in a cloud system. In addition, the integration of BG prediction based on LSTM into the system means that preventive alerts can be generated before critical hypoglycemic/hyperglycemic events occur. Thus, by knowing this information as early as possible, an individual can avoid the worst conditions in the future. Moreover, in a critical situation, a suggestion to visit the medical doctor can be shown by the system.

Other suggestions for diabetic patients are physical activity and exercise. Physical activity is defined as any movement that increases energy use, while exercise is a subset of physical activity that is more calculated or structured. Physical activity and exercise should be recommended to all diabetic patients as part of managing BG and improving health. A report published in Diabetes Care includes a recommendation for T1D and T2D patients. T2D patients should engage in 150 min or more of moderate-to-vigorous activity weekly. The activity should be carried out for at least three days/week, with no more than two consecutive days without activity. Walking, leg extensions, or overhead arm stretches every 30 min during a prolonged period of being sedentary are suggested to improve blood sugar management, particularly for patients with T2D. For T1D, activity is suggested to the youth and adults. Children and adolescents with T1D or T2D should engage in 60 min/day or more of moderate or vigorous aerobic activity with vigorous muscle and bone strengthening activities at least three days/week [79]. Furthermore, weight loss is an important goal for overweight or obese persons, particularly those with T2D. A report by the ADA shows the benefits of weight loss for the prevention and management of T2D. The results show that weight loss can be achieved by a lifestyle change, which includes a reduction in energy intake and an increase in physical activity. The benefit of weight loss reduces CVD risk, improves glycemic control, and can prevent the development of T2D for those with prediabetes. The report maintained that by applying a moderate decrease in caloric balance (500–1000 kcal/day) progressive weight loss (1–2 lb/week) can be achieved. In addition, for most patients, weight loss diets should supply at least 1000–1200 kcal/day for women and 1200–1600 kcal/day for men [80].

**Figure 10:**
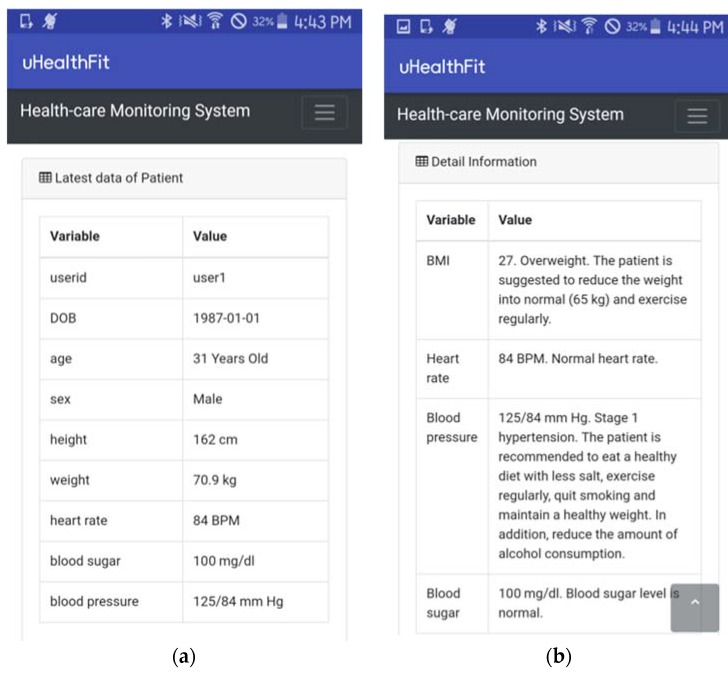
(**a**) The healthcare app showing the current user’s health data and (**b**) healthcare suggestions.

The aforementioned descriptions (physical activity and weight loss) can be combined with our proposed system (healthcare monitoring system, diabetes classification, and BG prediction) to improve the health quality of targeted individuals. Figure 10a shows an example of the app prototype presenting the latest vital signs data from a user. The current reading information from the BLE-based sensor devices such as weight, heart rate, BG level, and blood pressure is presented in the interface. In addition, personal data such as date of birth, sex, and height are also presented to the targeted user. The vital signs data of the user is presented in Figure 10b, such as the BMI, heart rate, blood pressure, and BG level. The app also presents several healthcare suggestions for the current user to improve his/her quality of health. The user is suggested to maintain a healthy diet, lose weight, and exercise regularly to improve his/her health quality.

## 5. Conclusions and Future Works

To the best of our knowledge, the present study is the first focusing on system integration of BLE-based sensor device, smartphone, real-time data processing and machine learning-based methods to predict diabetes and BG levels. The proposed model is expected to help users monitor their vital signs data from BLE-based sensor using their smartphone. Additionally, the proposed model helps users to discover the risk of diabetes at an early stage as well as help patients to obtain future predictions of their BG levels. Therefore, users can avoid the worst conditions in the future.

In this study, we showed that by integrating BLE-based sensor devices with the proposed personalized healthcare monitoring system, the complete history of a user’s vital signs data can be gathered and analyzed. The healthcare monitoring system should be scalable to accommodate the growing volume of sensor data from BLE-based sensor devices and the number of patients. Thus, real-time data processing that utilizes Apache Kafka and NoSQL MongoDB was proposed in this study. The experimental results show that the throughput and latency of the system were affected by the size of sensor documents and number of clients. In addition, the proposed real-time data processing allowed the healthcare monitoring system to process a huge amount and continuous user sensor data more efficiently compared to the traditional model. Furthermore, the performance of the BLE-based sensors was also analyzed with various measurements such as the average data packet delivery and CPU/memory usage of the android app. The data packet delivery performance was affected by the distance between the BLE-based sensor and the smartphone, while the CPU and memory usage were affected by the data transmission to the server. However, for all the experimental scenarios, the BLE-based sensor devices successfully gathered and transmitted the vital signs data of the user to the smartphone within an acceptable distance. In addition, the current commercially available smartphones are sufficiently capable of performing as gateways for transmitting sensor data to the server since the app only required low CPU and memory usage.

We propose diabetes classification and BG prediction given the input of the user vital signs data gathered by BLE-based sensor devices. The classification algorithm based on MLP was used to classify diabetes patients, while LSTM was utilized to predict BG. Both prediction models (MLP and LSTM) showed significant results by providing high accuracy and low error compared to the other models tested. Two types of user are expected to utilize the results of this study: normal individuals (currently not diagnosed with diabetes) and diabetic patients. Normal individuals can utilize the proposed healthcare monitoring system for early checking of their current health status as well as predicting whether they will develop diabetes in the future, while the proposed system can be used as a personal tool to control/ self-manage diabetic patients’ chronic condition. Furthermore, diabetic patients can obtain future predictions of their BG and, by knowing this information as early as possible, can avoid the worsening of their condition in the future.

The dataset for the diabetes classification in this study was limited to PIMA Indian women, so it is difficult to generalize the robust classification model to be applied for different purposes. If a real dataset is collected from a real-case implementation, it will increase the accuracy of the classification model.

## Figures and Tables

**Table 1:** Performance metrics for the classification model.

Performance Metric	Formula
Precision	TP/(TP+FP)
Recall	TP/(TP+FN)
Accuracy	(TP+TN)/(TP+TN+FP+FN)

**Table 2:** The performance comparison of the classifiers for diabetes classification.

Method	Precision (%)	Recall (%)	Accuracy (%)
Random Forest	72.7	73	73.046
NB	76.1	76.7	76.6927
SVM	76	76.6	76.562
Logistic Regression	75.4	76.0	76.0417
MLP	**76.6**	**77.1**	**77.083**

**Table 3:** The performance metrics for the forecasting model.

Performance Metric	Formula
Correlation coefficient (*r*)	$\frac{\sum_{i=1}^{n}\left(y_{i}-{\bar{y}}_{i}\right)\left({\hat{y}}_{i}-{\bar{\hat{y}}}_{i}\right)}{\sqrt{\sum_{i=1}^{n}{\left(y_{i}-{\bar{y}}_{i}\right)}^{2}}\sqrt{\sum_{i=1}^{n}{\left({\hat{y}}_{i}-{\bar{\hat{y}}}_{i}\right)}^{2}}}$
RMSE	$\sqrt{\frac{1}{n}\overset{n}{\underset{i=1}{\sum }}{\left(y_{i}-{\hat{y}}_{i}\right)}^{2}}$

**Table 4:** The model comparison for forecasting BG.

Dataset	Method	RMSE	*r*
Dataset 1	LSTM	**25.621**	**0.647**
	Linear Regression	44.069	−0.019
	Moving Average	47.487	−0.183
Dataset 2	LSTM	**2.285**	**0.999**
	Linear Regression	82.592	−0.071
	Moving Average	42.946	0.710
